# Growth Differentiation Factor-15 Predicts Major Bleeding in Cancer Patients: Results From the Vienna CAT-BLED Study

**DOI:** 10.1016/j.jaccao.2024.11.007

**Published:** 2025-01-14

**Authors:** Cornelia Englisch, Stephan Nopp, Florian Moik, Daniel Steiner, Angelika M. Starzer, Monika Fritzer-Szekeres, Matthias Preusser, Anna S. Berghoff, Ingrid Pabinger, Cihan Ay

**Affiliations:** a Division of Hematology and Hemostaseology, Department of Medicine I, Medical University of Vienna, Vienna, Austria; b Division of Oncology, Department of Internal Medicine, Medical University of Graz, Graz, Austria; c Division of Oncology, Department of Medicine I, Medical University of Vienna, Vienna, Austria; d Christian Doppler Laboratory for Personalized Immunotherapy, Department of Medicine I, Medical University of Vienna, Vienna, Austria; e Department of Laboratory Medicine, Medical University of Vienna, Vienna, Austria

**Keywords:** biomarkers, bleeding, cancer, hemorrhage, major bleeding, risk prediction

## Abstract

**Background:**

The hemostatic system is tightly interconnected with cancer. Research has focused predominantly on thrombotic complications, but less is known about bleeding and bleeding risk prediction. Growth differentiation factor (GDF)-15 has previously emerged as a prognostic biomarker for bleeding.

**Objectives:**

The aim of this study was to investigate the association and predictive ability of GDF-15 for bleeding risk in patients with cancer.

**Methods:**

The CAT-BLED (Vienna Cancer, Thrombosis, and Bleeding) study is a prospective, observational cohort study including cancer patients initiating systemic anticancer therapies. Patients were followed for up to 2 years for thrombotic and bleeding events. The primary outcome was major bleeding. GDF-15 was measured at inclusion and at 3 and 6 months of follow-up.

**Results:**

A total of 779 patients (48% women, median age 62 years, 15% on therapeutic anticoagulation) were included. During a median follow-up period of 18 months, 79 patients (10.1%) experienced major bleeding (12-month cumulative incidence 8.8%; 95% CI: 6.7-10.9). Higher GDF-15 levels were independently associated with increased major bleeding risk (adjusted subdistribution HR per doubling: 1.29; 95% CI: 1.04-1.59), and patients with levels greater than the cohort median (1,864 ng/L) had a significantly higher 12-month cumulative incidence (13.1% vs 4.6%; *P* < 0.001). This association remained robust in follow-up measurements at 3 and 6 months. GDF-15 showed moderate to good discrimination for predicting 6-month major bleeding risk (C statistic = 0.69; 95% CI: 0.60-0.77). GDF-15 was not associated with venous thromboembolism but was strongly associated with mortality (adjusted HR: 1.37; 95% CI: 1.25-1.50).

**Conclusions:**

GDF-15 levels predict major bleeding risk in cancer patients and are not associated with venous thromboembolism, making GDF-15 a particularly promising biomarker for bleeding risk prediction.

Cancer and the hemostatic system are closely interconnected, often resulting in hemostatic imbalances in patients with cancer. These imbalances may manifest as thrombotic complications, hemorrhagic complications, or both.^1^ Consequently, patients with cancer face an increased risk for venous thromboembolism (VTE), arterial thromboembolic events, and bleeding.^2^, ^3^, ^4^, ^5^, ^6^ Although research has focused primarily on the risk for thrombotic events, the pathophysiology and risk factors for bleeding events in cancer patients remain less understood. This knowledge gap makes it particularly challenging to identify patients at high risk for bleeding.

Current guidelines recommend assessing individual VTE risk in patients with cancer to guide primary thromboprophylaxis.^7^^,^^8^ In contrast, bleeding risk assessment remains significantly limited because of a lack of data, particularly on bleeding risk prediction. Established bleeding risk models, which are derived primarily from noncancer populations, have shown poor performance in cancer patients.^9^^,^^10^ Although various models and biomarkers exist for predicting thrombosis and its recurrence in the general population,^11^ no prognostic biomarker for bleeding has been established in cancer patients. For effective individualized risk assessment, however, both thrombotic and bleeding risks must be considered.

Growth differentiation factor (GDF)-15 is a stress-response protein belonging to the transforming growth factor-β superfamily and is present in most human tissues. In healthy individuals, GDF-15 is expressed at low levels but increases in response to stress.^12^ Furthermore, evidence suggests that GDF-15 plays a role in tumorigenesis and disease progression in cancer patients.^12^ To date, GDF-15 has been most extensively studied in cardiovascular diseases, particularly in patients with atrial fibrillation (AF). Research indicates that GDF-15 is a valuable biomarker for cardiovascular events, all-cause mortality, and bleeding in patients with AF.^13^, ^14^, ^15^ Consequently, GDF-15 has been incorporated into bleeding and mortality risk prediction models, such as the ABC risk scores.^14^ Additionally, several studies have reported promising results for GDF-15 in predicting bleeding risk in other populations, including a small cohort of cancer patients.^16^, ^17^, ^18^, ^19^

Notably, our previous research demonstrated that GDF-15 does not predict VTE risk.^20^ Given its potential to differentiate between bleeding and thrombotic risk, a thorough investigation into the role of GDF-15 in predicting bleeding in cancer patients is warranted. Therefore, we aimed to evaluate the ability of GDF-15 to predict bleeding risk in cancer patients initiating systemic anticancer therapies.

## Methods

### Study design and population

This study was conducted as part of the CAT-BLED (Vienna Cancer, Thrombosis, and Bleeding) study, a single-center, prospective, observational cohort study at the Medical University of Vienna. The aim of the CAT-BLED study is to investigate the risk and risk factors for VTE and bleeding in cancer patients starting new systemic anticancer therapies.^21^

Consecutive adult patients referred to the oncology day clinic, a clinic patients attend to receive systemic anticancer therapies, were eligible for inclusion if they had histologically confirmed cancer. This included patients with newly diagnosed cancer as well as those with recurrent or progressive cancer after prior anticancer therapies. Exclusion criteria were inability or refusal to provide informed consent and age <18 years.

Patients included in this analysis were recruited between July 2019 and March 2023. Baseline blood samples were collected prior to the first administration of systemic anticancer therapy at the oncology day clinic upon study enrollment, with additional samples collected at 3 months and 6 months. Biobanking was performed within the Translational Research Unit Biobanking Program for Personalized Immunotherapy (approval number EK 1164/2019) of the Division of Oncology at the Medical University of Vienna.

Patients were followed in person during routine visits for up to 2 years, and all reported events were cross-validated by screening electronic medical records. The study was approved by the ethics committee of the Medical University of Vienna (approval number EK 1533/2019), and all patients provided written informed consent. This report adheres to the Strengthening the Reporting of Observational Studies in Epidemiology guidelines.^22^

### Blood sampling and biomarker measurements

Venous blood samples were collected into serum clot activator tubes (Vacuette, Greiner Bio-One) using sterile venipuncture. Samples were centrifuged to obtain serum, and biomaterial was processed and stored following standard operating procedures by the MedUni Wien Biobank in an ISO 9001:2015–certified environment until analysis.^23^ All samples were coded before laboratory analysis, and technicians remained blinded to patients’ characteristics throughout.

GDF-15 levels were measured using the Elecsys GDF-15 assay (Roche Diagnostics) on the cobas e 801 module (Roche Diagnostics) in the Department of Laboratory Medicine at the Medical University of Vienna.

### Outcomes of interest

The primary outcome of interest was major bleeding, defined according to the International Society on Thrombosis and Haemostasis criteria as overt bleeding that met at least 1 of the following criteria: 1) associated with a decrease in hemoglobin level of ≥2 g/dL; 2) required transfusion of ≥2 U packed red cells; 3) occurred at a critical site; or 4) contributed to death.^24^

The severity of major bleeding was further stratified on the basis of clinical management as follows: category 1, not considered a clinical emergency; category 2, requires treatment but is not considered a clinical emergency; category 3, clinical emergency (eg, hemodynamic instability, intracranial bleeding with neurologic symptoms); and category 4, bleeding that led to death before or almost immediately after presentation to the hospital.^25^ All major bleeding events were adjudicated by an independent committee comprising experts in hemostaseology.

The secondary outcomes of interest included clinically relevant bleeding, defined as major bleeding and clinically relevant nonmajor bleeding per International Society on Thrombosis and Haemostasis criteria^24^; VTE, including deep vein thrombosis, pulmonary embolism, visceral vein thrombosis, catheter-related thrombosis, and symptomatic superficial vein thrombosis of the lower limb with at least 5 cm extension; and all-cause mortality.^21^

### Statistical analysis

Statistical analyses were conducted using SPSS version 28.0 (IBM SPSS Statistics) and RStudio version 4.2.0 (R Studio) and were predefined in the statistical analysis plan (see the Supplemental Appendix). The alpha level was set at 0.05. Standard summary statistics were applied to describe patients’ baseline characteristics, including absolute frequencies, percentages, median, and 25th to 75th percentiles (Q1-Q3). For visualization, patients were stratified by median GDF-15 level.

Cumulative incidence estimates of bleeding events with 95% CIs were calculated, accounting for mortality as a competing risk, and comparisons were made using Gray’s test. Median follow-up time was determined using the reverse Kaplan-Meier method.

GDF-15 levels were log_2_ transformed to address their skewed distribution. The association of GDF-15 with bleeding was assessed using univariable and multivariable Gray’s proportional subhazard regression models, accounting for the competing risk for death.^26^ Model assumptions were verified by testing for time-dependent covariate effects to assess proportionality and applying spline functions to evaluate linearity. The association between GDF-15 levels and all-cause mortality was analyzed using Cox regression.

Results are reported as HRs and subdistribution HRs (SHRs) with 95% CIs, representing the correlation between log_2_-transformed GDF-15 levels and outcome events and reflecting the increase in hazard for each doubling of GDF-15 levels. To investigate potential nonlinear associations between GDF-15 levels and outcomes, a restricted cubic splines analysis with 4 knots was performed.

The prognostic value of GDF-15 for predicting major bleeding risk was compared with that of established bleeding risk scores for the general population (HAS-BLED^27^ and CAT-BLEED^9^) using several metrics. Overall model performance was assessed using the Brier score, which measures the accuracy of probabilistic predictions. Discrimination was evaluated using Harrell’s C statistic for logistic regression to assess 6-month major bleeding risk, and calibration was analyzed using calibration plots comparing predicted vs observed 6-month risk.

The incremental value of adding GDF-15 to these scores was assessed using model comparison measures, including net reclassification index, integrated discrimination improvement, likelihood ratio testing, adequacy index, and fraction of new information. CIs at the 95% level were calculated from bootstrapped values for all metrics assessing model performance.

## Results

### Patient characteristics

A total of 779 patients were included in the study, 47.9% of whom were women, with a median age of 62 years (Q1-Q3: 54-70 years). Approximately one-half of the patients (49.6%) had newly diagnosed cancer, and 69.2% presented with stage IV disease at study inclusion. The 3 most common tumor types were lung (22.5%), head and neck (11.0%), and pancreatic (10.5%) cancer.

The most frequently initiated systemic anticancer therapies after study inclusion were chemotherapy (46.2%), immune checkpoint inhibitor therapy (15.3%), and a combination of both therapies (16.8%). At study inclusion, 117 patients (15.0%) were receiving therapeutic anticoagulation, and 125 patients (16.0%) were on antiplatelet therapy. Detailed patient characteristics are summarized in Table 1, and the study flowchart is provided in Supplemental Figure 1.

**Table 1:** Patient Characteristics (N = 779)

Age, y	62 (54-70)
Female	373 (47.9)
BMI, kg/m^2^	24 (21-27)
Tumor type	
Lung	175 (22.5)
Head and neck	86 (11.0)
Pancreatic	82 (10.5)
Breast	75 (9.6)
Sarcoma	59 (7.6)
Colorectal	60 (7.7)
Other	59 (7.6)
Brain	50 (6.4)
Hepatobiliary	36 (4.6)
Stomach	35 (4.5)
Esophageal	26 (3.3)
Urinary	15 (1.9)
Lymphoma	13 (1.7)
Prostate	6 (0.8)
Gynecologic	2 (0.3)
Newly diagnosed cancer	386 (49.6)
Stage IV vs stages I, II, and III	539 (69.2)
Systemic therapy after study inclusion	
Chemotherapy	360 (46.2)
Chemotherapy + ICI therapy	131 (16.8)
ICI therapy	119 (15.3)
Targeted therapy + chemotherapy	92 (11.8)
Targeted therapy	35 (4.5)
No systemic therapy	26 (3.3)
Targeted therapy + ICI therapy	15 (1.9)
TKI	1 (0.1)
Diabetes mellitus	96 (12.3)
Arterial hypertension	270 (34.7)
Atrial fibrillation	46 (5.9)
History of venous thromboembolism	98 (12.6)
History of arterial thromboembolism	70 (9.0)
Therapeutic anticoagulation^a^	117 (15.0)
Antiplatelet therapy	125 (16.0)
ECOG performance status^b^	
0	496 (65.0)
1	209 (27.4)
2	53 (6.9)
3	4 (0.5)

Values are median (Q1-Q3) or n (%). Baseline data were available in all patients.

BMI = body mass index; ECOG = Eastern Cooperative Oncology Group; ICI = immune checkpoint inhibitor; TKI = tyrosine kinase inhibitor.

a Defined as anticoagulation for venous thromboembolism treatment or anticoagulation for stroke prevention in atrial fibrillation or anticoagulation in patients with mechanical heart valves or vascular grafts.

b Missing in 17 patients.

During a median observation period of 18 months (Q1-Q3: 11-24 months), 79 patients (10.1%) experienced first major bleeding events. The cumulative incidence of major bleeding at 6, 12, and 24 months was 5.5% (95% CI: 3.8%-7.1%), 8.8% (95% CI: 6.7%-10.9%), and 12.2% (95% CI: 9.6%-14.9%), respectively (Supplemental Figure 2). Most major bleeding events were classified as grade 2 in severity (69.7%) and were located primarily in the upper gastrointestinal tract (35.4%). Tumor-related bleeding accounted for 43.0% of major bleeding events, and 8 events (10.1%) were fatal (Table 2). During the same observation period, 349 patients (44.8%) died, with cumulative all-cause mortality incidences at 6, 12, and 24 months of 20.0% (95% CI: 17.1%-22.8%), 34.7% (95% CI: 31.1%-38.1%), and 58.0% (95% CI: 53.2%-62.2%), respectively. Two patients (0.3%) were lost to follow-up.

**Table 2:** Characteristics of First Major Bleeding Events (N = 79)

Clinical severity	
1	5 (6.3)
2	55 (69.7)
3	11 (13.9)
4	8 (10.1)
Location	
Upper gastrointestinal	28 (35.4)
Lower gastrointestinal	15 (19.0)
Intracranial	15 (19.0)
Oropharyngeal	10 (12.7)
Urinary tract	4 (5.1)
Epistaxis	3 (3.8)
Other^a^	4 (5.1)
Primary cancer type	
Head and neck	15 (19.0)
Pancreas	13 (16.5)
Lung	13 (16.5)
Breast	6 (7.6)
Colorectal	6 (7.6)
Esophageal	6 (7.6)
Stomach	3 (3.8)
Hepatobiliary	3 (3.8)
Brain	2 (2.5)
Sarcoma	2 (2.5)
Other^b^	10 (12.7)
Tumor bleeding	34 (43.0)
Tumor bleeding location	
Intracranial	13 (38.2)
Oropharyngeal	10 (2.9)
Upper gastrointestinal	7 (20.6)
Urinary tract	2 (5.9)
Pulmonary	1 (2.9)
Peritoneal	1 (2.9)
Therapeutic anticoagulation^c^	28 (35.4)
Prophylactic anticoagulation^c^	10 (12.7)
Antiplatelet therapy^c^	14 (17.7)
Fatal	8 (10.1)

Values are n (%).

a Pericardial, peritoneal, and pulmonary.

b Including melanoma (n = 1), mesothelioma (n = 3), cancer of unknown primary (n = 2), thyroid cancer (n = 1), neuroendocrine carcinoma (n = 1), anal carcinoma (n = 1), and hemangioblastoma (n = 1).

c At the time of bleeding.

### GDF-15 levels and associations with clinical and laboratory parameters at baseline

The median GDF-15 serum level in the cohort was 1,864 ng/L (Q1-Q3: 1,061-3,490 ng/L). GDF-15 measurements failed in 3 patients (0.4%). The distribution of GDF-15 levels stratified by tumor type is shown in Supplemental Figure 3. Patients with hepatobiliary cancer exhibited the highest GDF-15 levels (median 6,233 ng/L; Q1-Q3: 3,285-9,861 ng/L), whereas patients with brain tumors had the lowest levels (median 858 ng/L; Q1-Q3: 522-1,150 ng/L).

In a multivariable model including variables measured at study inclusion, higher GDF-15 levels were most strongly associated with higher gamma-glutamyl transferase, lower hemoglobin, higher age, higher lactate dehydrogenase, higher glutamic oxaloacetic transaminase, and stage IV disease, ranked by their relative effects on model output. However, no single variable explained more than 10% of the variation in GDF-15 levels (Supplemental Figure 4).

### GDF-15 and its association with major bleeding

**Figure 1:**
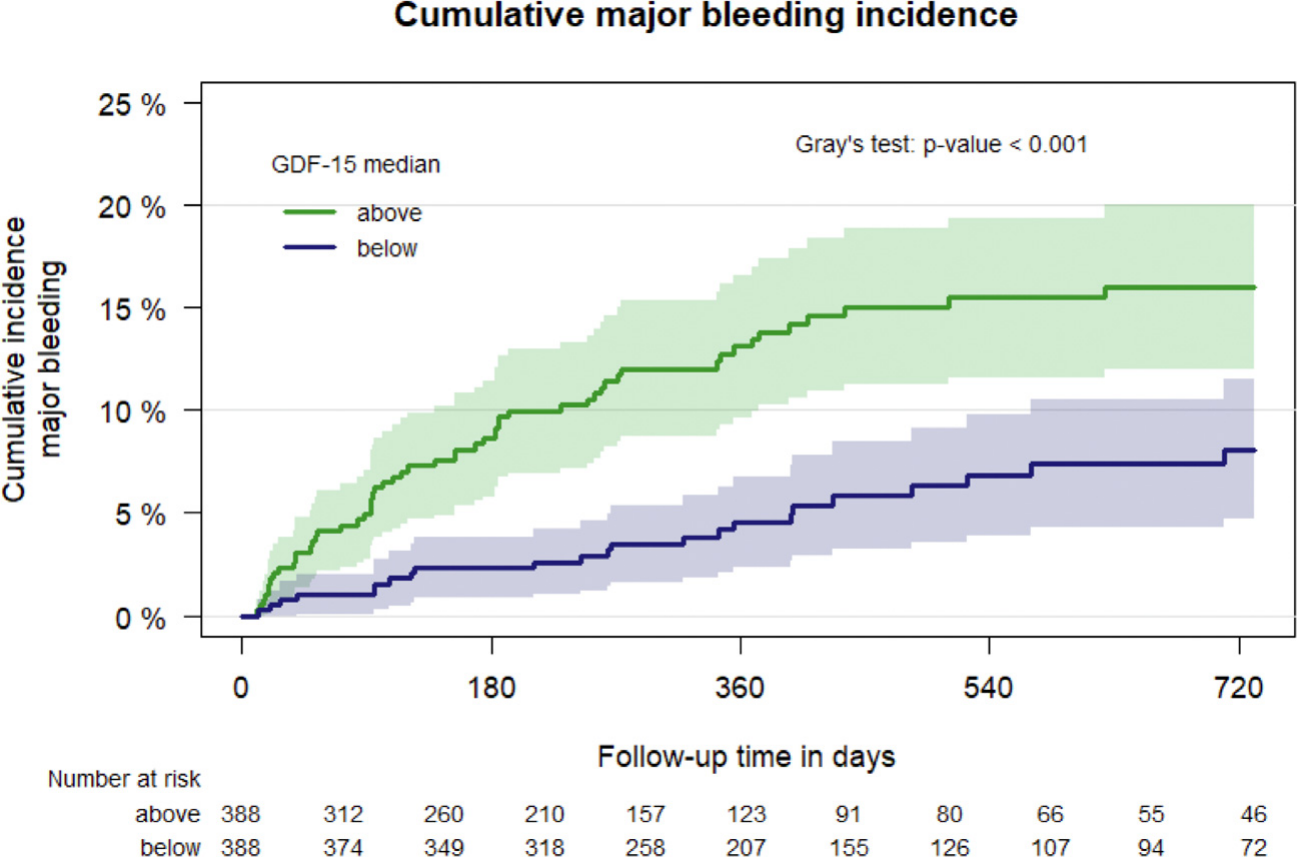
Cumulative Incidence of Major Bleeding According to GDF-15 Level Patients were stratified by growth differentiation factor (GDF)-15 level, with the group higher than 1,864 ng/L (the median value) compared with the group at or lower than 1,864 ng/L using Gray’s test for competing risks.

Elevated GDF-15 levels were significantly associated with an increased risk for major bleeding (univariable SHR per doubling: 1.42; 95% CI: 1.23-1.64). Stratifying the cohort at the median GDF-15 level (1,864 ng/L) revealed a substantially higher risk for major bleeding in patients with levels higher than this cutoff compared with those with levels lower than the cutoff throughout the observation period (Figure 1). The 12-month cumulative incidence of major bleeding was 13.1% (95% CI: 9.6%-16.6%) in patients with GDF-15 levels higher than the median vs 4.6% (95% CI: 2.4%-6.8%) in those with levels lower than the median.

**Figure 2:**
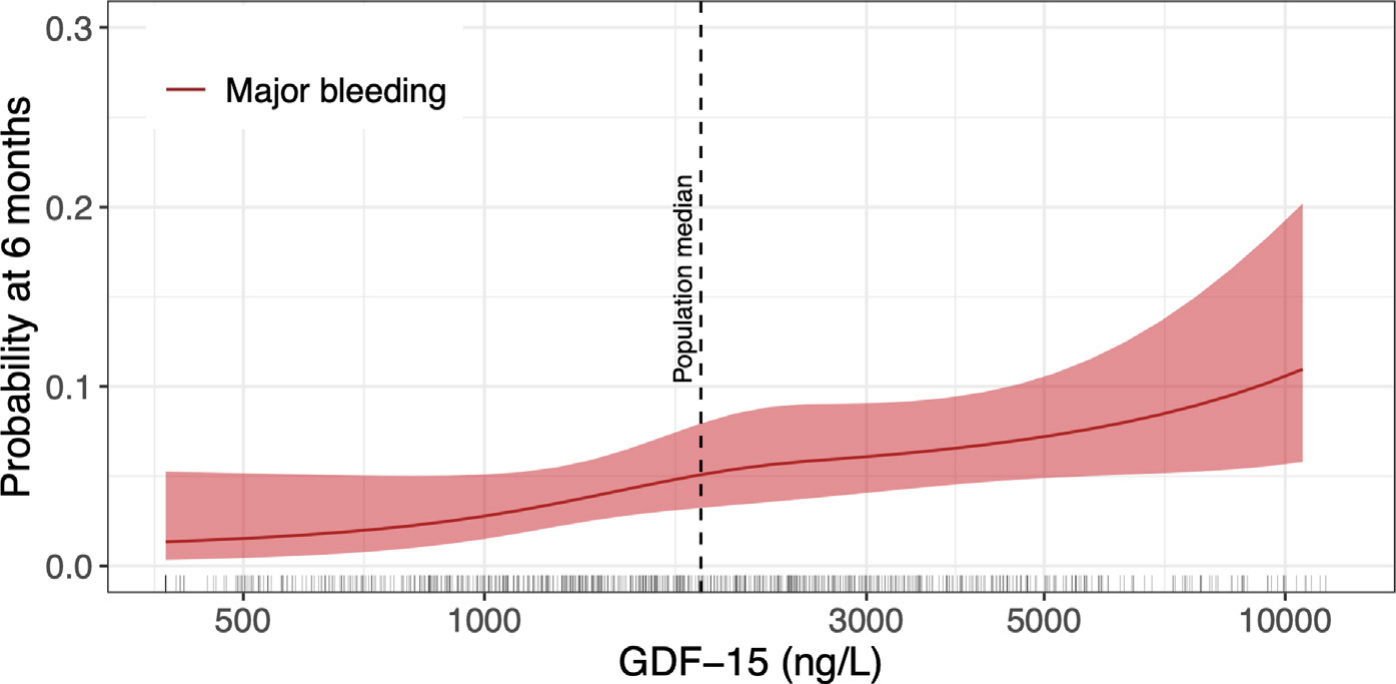
Six-Month Predicted Probabilities of Major Bleeding in Relation to Continuous GDF-15 Levels This analysis was based on Gray’s model incorporating growth differentiation factor (GDF)-15 with restricted cubic splines and accounting for all-cause death as a competing risk. The x-axis was log_10_ transformed. Predicted probabilities are shown up to the 95th percentile of GDF-15 levels, which range from 400 to 59,707 ng/L. The dashed line represents the cohort median GDF-15 level.

The probability of major bleeding over 6 months of observation, relative to continuous baseline GDF-15 level, is shown in Figure 2. The data indicate a log-linear relationship between GDF-15 level and the risk for major bleeding.

**Figure 3:**
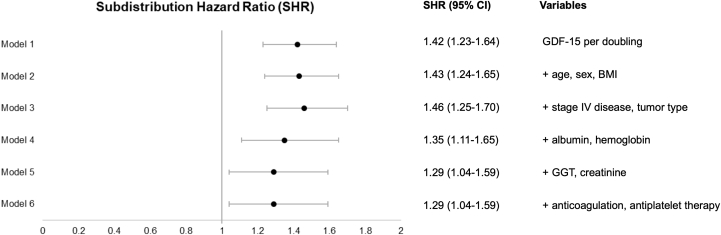
Association of GDF-15 With Major Bleeding in Univariable and Multivariable Analyses Subdistribution HRs (SHRs) are displayed for log_2_-transformed levels of growth differentiation factor (GDF)-15. The SHR represents the risk increase associated with each doubling of GDF-15 level. All-cause death was considered a competing risk. Patients with head and neck cancer were classified as high risk for bleeding, and adjustment for head and neck vs other tumor types was performed. BMI = body mass index; GGT = gamma-glutamyl transferase.

The independent association between GDF-15 and major bleeding was confirmed in multivariable analyses (Figure 3). After adjusting for patient demographics, tumor type, stage IV disease, laboratory parameters, and antithrombotic therapy, the association remained strong and robust (adjusted SHR per doubling: 1.29; 95% CI: 1.04-1.59).

### GDF-15 and its prognostic value for major bleeding

The prognostic value of GDF-15 for predicting major bleeding was evaluated using 2 common bleeding risk models: HAS-BLED and CAT-BLEED. Comparative statistical indexes are displayed in Table 3. Both models demonstrated poor discriminatory performance for predicting major bleeding at 6 months (for HAS-BLED, C statistic = 0.51 [95% CI: 0.45-0.58]; for CAT-BLEED, C statistic = 0.54 [95% CI: 0.47-0.63]). However, incorporating GDF-15 levels significantly improved the discrimination of both models (for HAS-BLED plus GDF-15, C statistic = 0.68 [95% CI: 0.59-0.77]; for CAT-BLEED plus GDF-15, C statistic = 0.69 [95% CI: 0.61-0.77]), as indicated by integrated discrimination improvement, net reclassification index, and other measures of model comparison (Table 3).

**Table 3:** Predictive Performance of GDF-15 and Bleeding Risk Scores After Addition of GDF-15

	GDF-15 Only	CAT-BLEED Score	CAT-BLEED + GDF-15	HAS-BLED	HAS-BLED + GDF-15
Discrimination					
C statistic (95% CI)	0.69 (0.60-0.77)	0.54 (0.47-0.63)	0.69 (0.61-0.78)	0.51 (0.45-0.58)	0.68 (0.59-0.77)
Overall performance					
Brier score (95% CI)	0.050 (0.04-0.06)	0.051 (0.04-0.07)	0.050 (0.04-0.06)	0.051 (0.04-0.06)	0.050 (0.04-0.06)
Likelihood function					
Model likelihood ratio test	17.54 (*P* < 0.001)	0.30 (*P* = 0.59)	17.55 (*P* < 0.001)	0.13 (*P* = 0.72)	15.84 (*P* < 0.001)
Measures of model comparisons					
NRI (95% CI)		—	0.515 (0.21-0.82) (*P* = 0.001)	—	0.510 (0.20-0.82) (*P* = 0.001)
IDI (95% CI)		—	0.027 (0.01-0.04) (*P* = 0.001)	—	0.027 (0.01-0.04) (*P* = 0.001)
Likelihood ratio test for 2 nested models		—	1.725 (*P* < 0.001)	—	1.571 (*P* < 0.001)
Adequacy index		—	0.02	—	0.01
Fraction of new information from GDF-15		—	0.99	—	0.99

Statistical indexes for GDF-15 and the additive value of GDF-15 to predict 6-month risk for major bleeding in patients with cancer. The CAT-BLEED and HAS-BLED scores are clinical prediction tools used to assess bleeding risk. The C statistic measures a model’s ability to discriminate between patients who will experience major bleeding and those who will not. Values range from 0.5 (no discrimination) to 1.0 (perfect discrimination), with higher values indicating better predictive discrimination. The Brier score, ranging from 0 to 1, evaluates the accuracy of probabilistic predictions by calculating the mean squared difference between predicted probabilities and actual outcomes; lower Brier scores indicate better model performance. The added value of GDF-15 to both models was assessed using several measures of model comparison. The continuous NRI represents the sum of the net percentages of individuals with and without major bleeding who were correctly reassigned to different predicted risk categories when GDF-15 was added to the models (range: −2 to 2). IDI is defined as the difference in discrimination slopes between models with and without GDF-15. The fraction of new information, proposed as the *R*^2^ measure specifically for binary outcomes, reflects the proportion of total predictive information added by log-transformed GDF-15 to the CAT-BLEED and HAS-BLED scores. Ninety-five percent CIs were calculated using bootstrapped values.

GDF = growth differentiation factor; IDI = integrated discrimination improvement; NRI = net reclassification index.

A model using only GDF-15 also showed good discriminatory performance for predicting the 6-month risk for major bleeding (C statistic = 0.69; 95% CI: 0.60-0.77). Similar improvements were observed in calibration performance. Both established models showed extremely poor calibration in this cohort, with substantial discrepancies between observed and predicted bleeding risks. However, the addition of GDF-15 significantly improved calibration, as demonstrated by the calibration plots (Supplemental Figure 5).

### GDF-15 and its association with clinically relevant bleeding

To complement our primary analysis of major bleeding, we conducted a secondary analysis of the composite outcome of major bleeding and clinically relevant nonmajor bleeding, collectively referred to as clinically relevant bleeding. Elevated GDF-15 levels were significantly associated with an increased risk for clinically relevant bleeding (univariable SHR per doubling: 1.29; 95% CI: 1.16-1.44) (Supplemental Figure 6).

Additionally, plotting the probability of clinically relevant bleeding over a 6-month period against continuous baseline GDF-15 levels revealed again a log-linear relationship, indicating that as GDF-15 levels increase, the risk for clinically relevant bleeding rises proportionally (Supplemental Figure 7). After adjusting for demographics, tumor type, stage IV disease, laboratory parameters, and antithrombotic therapy, the association remained robust (adjusted SHR per doubling: 1.35; 95% CI: 1.11-1.65) (Supplemental Figure 8).

### GDF-15 and its association with VTE and all-cause mortality

GDF-15 levels were not associated with VTE in either unadjusted analyses (crude SHR per doubling: 0.99; 95% CI: 0.85-1.15) or after adjusting for potential confounders (adjusted SHR: 1.09; 95% CI: 0.89-1.32). Detailed data on the analyses of VTE can be found in Supplemental Figures 9 and 10.

**Figure 4:**
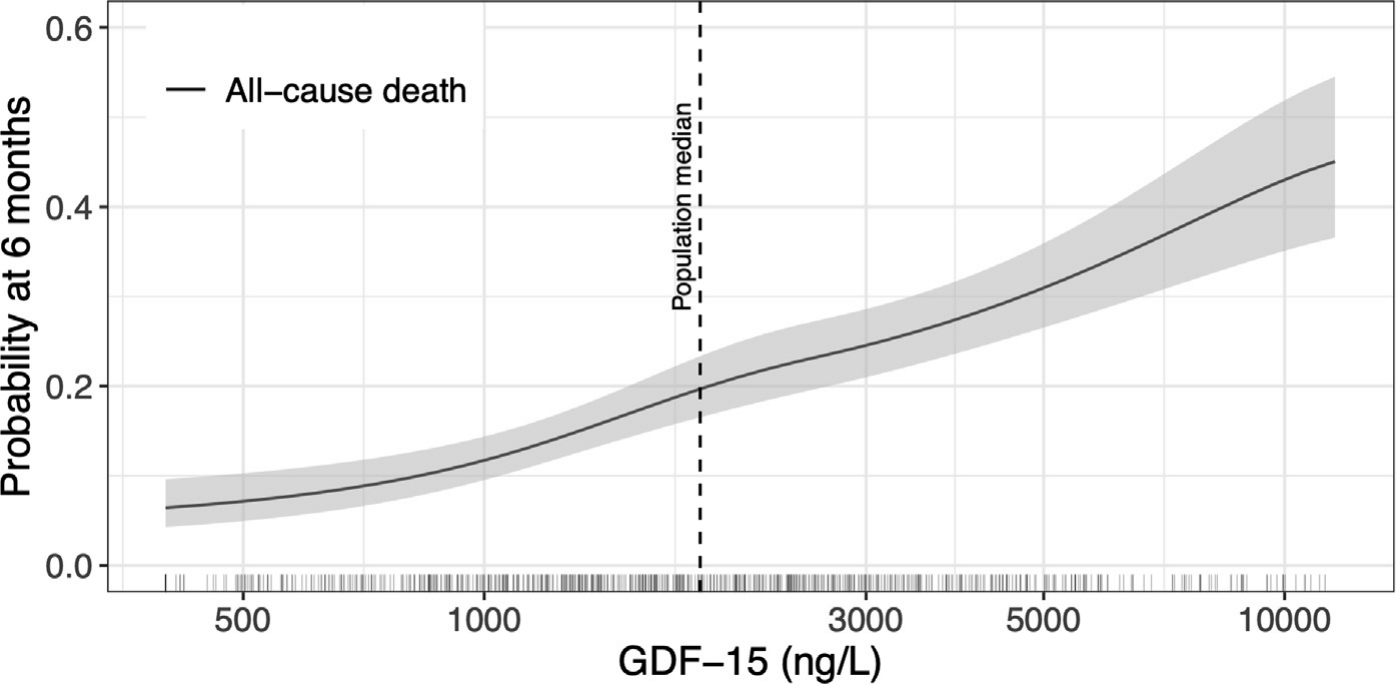
6-Month Predicted Probabilities of All-Cause Death in Relation to Continuous GDF-15 Levels The x-axis was log_10_ transformed. Predicted probabilities are shown up to the 95th percentile of growth differentiation factor (GDF)-15 levels, which range from 400 to 59,707 ng/L. The dashed line represents the cohort median GDF-15 level.

**Figure 5:**
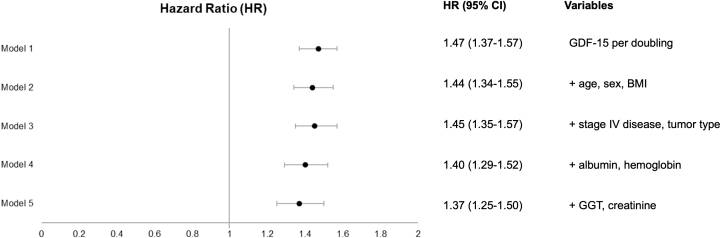
Association of GDF-15 With All-Cause Mortality in Univariable and Multivariable Analyses HRs are displayed for log_2_-transformed levels of GDF-15. The hazard ratio represents the risk increase associated with each doubling of GDF-15 levels. Abbreviations as in Figure 3.

In contrast, elevated GDF-15 levels were strongly associated with all-cause mortality (crude HR: 1.47; 95% CI: 1.37-1.57). Mortality rates at 6 months, visualized in relation to continuous GDF-15 levels (Figure 4), indicate a log-linear increase in the risk for death with rising GDF-15 levels. After adjusting for confounders, including patient demographics, tumor-specific variables, and laboratory values, the strong relationship between GDF-15 and all-cause mortality persisted (adjusted HR: 1.37; 95% CI: 1.25-1.50) (Figure 5). The corresponding C statistic for GDF-15 of 0.71 (95% CI: 0.67-0.75) indicates good discriminatory performance for predicting all-cause mortality.

### Exploratory analyses on longitudinal GDF-15 measurements

Follow-up samples were available for 505 patients at 3 months and 334 patients at 6 months. The median change in GDF-15 level from baseline to the first follow-up was 340.5 ng/L (Q1-Q3: −58.0 to 1,236.0 ng/L) and from baseline to the second follow-up was 386.0 ng/L (Q1-Q3: −141.0 to 1,331.0 ng/L).

In patients alive at the 3-month follow-up, GDF-15 levels remained strongly associated with major bleeding (univariable SHR: 1.53; 95% CI: 1.28-1.82), and this association persisted at 6-month follow-up (univariable SHR: 1.35; 95% CI: 1.07-1.70). Similarly, GDF-15 levels showed a strong and robust association with all-cause mortality in patients alive at 3 months (univariable HR: 1.44; 95% CI: 1.29-1.60) and at 6 months (univariable HR 1.45; 95% CI 1.27-1.66).

## Discussion

In this prospective cohort study of 779 cancer patients, we evaluated the prognostic potential of GDF-15 as a biomarker for major bleeding (Central Illustration). Elevated GDF-15 levels were independently associated with an increased risk for major bleeding. Analyses of the predictive ability of GDF-15 revealed good performance in both discrimination and calibration for predicting 6-month major bleeding in this cancer cohort. Consistent with our previous study,^20^ GDF-15 was strongly associated with the risk for death but showed no association with VTE.

**Central Illustration:**
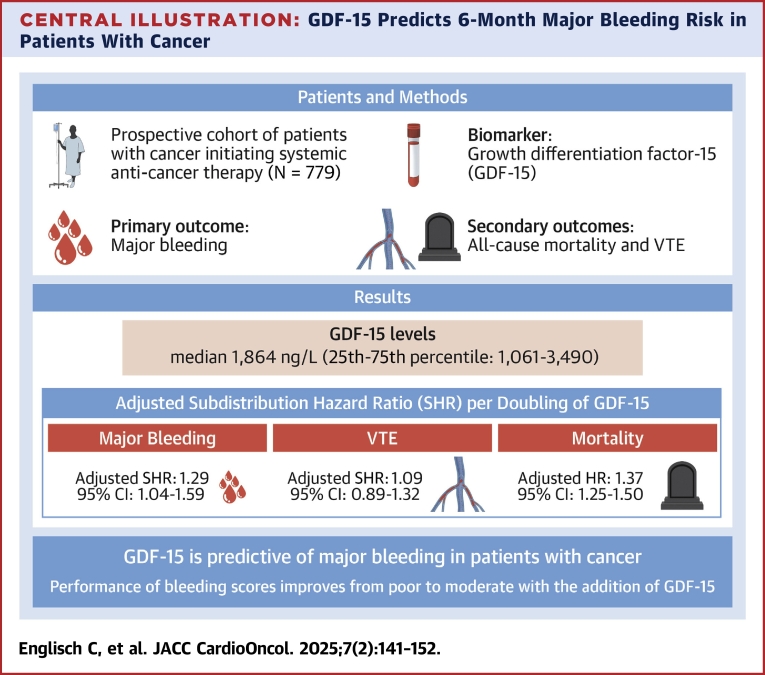
GDF-15 Predicts 6-Month Major Bleeding Risk in Patients With Cancer This illustration summarizes the study cohort, outcomes of interest, and key results of the analyses on growth differentiation factor (GDF)-15 in cancer patients. GDF-15 demonstrated an independent association with major bleeding risk and all-cause mortality but showed no association with venous thromboembolism (VTE) risk. Evaluated bleeding risk scores, including HAS-BLED and CAT-BLEED, showed improved performance when GDF-15 was incorporated. GDF-15 alone effectively predicted 6-month major bleeding risk.

Effective tools for stratifying bleeding risk in cancer patients are currently lacking, which poses a significant challenge to achieving a balanced risk-benefit evaluation of anticoagulation strategies. Established bleeding scores have been shown to perform poorly in cancer patients.^9^^,^^10^ Most bleeding assessment models designed for the general population include cancer as a risk factor, broadly categorizing all cancer patients as high risk. This oversimplification limits precise risk stratification within the cancer patient population.

Recently, 2 bleeding risk scores were developed specifically for cancer patients receiving anticoagulation: CAT-BLEED^9^ and B-CAT.^28^ However, the B-CAT score has yet to undergo external validation,^28^ and the CAT-BLEED score has demonstrated poor performance in external validation cohorts.^29^

In our cohort, GDF-15 was significantly associated with bleeding risk, even after rigorous adjustment for various clinical, cancer-specific, and laboratory parameters. Notably, each doubling of GDF-15 level corresponded to a 40% increase in major bleeding risk, and the prognostic potential of GDF-15 was confirmed. The predictive power of GDF-15 for bleeding has been previously reported in various patient populations. The strongest evidence for its predictive value comes from patients with AF receiving oral anticoagulation, which led to its inclusion in the ABC bleeding risk score.^14^ Additionally, GDF-15 has been associated with bleeding in patients with chronic kidney disease, those with acute coronary syndrome, and those without cardiovascular disease.^17^, ^18^, ^19^ Furthermore, in a post hoc analysis of the AVERT (Apixaban for the Prevention of Venous Thromboembolism in Cancer Patients) trial, which assessed direct oral anticoagulants for primary VTE prevention in cancer patients, GDF-15 levels were associated with bleeding risk in the apixaban group, which included 235 patients and 5 major bleeding events.^16^

Our study confirms and expands these exploratory findings with robust data on bleeding in an unselected cohort of consecutive cancer patients, particularly as GDF-15 levels were not predictive of major bleeding in the placebo group of the AVERT trial. We further assessed the predictive performance of the CAT-BLEED^9^ and HAS-BLED^27^ scores, the latter being one of the most commonly used bleeding risk prediction tools in general. Notably, both models were explicitly developed for patients receiving anticoagulation. However, the HAS-BLED score was not designed for cancer patients, and the CAT-BLEED score was derived from a randomized controlled trial population of cancer patients receiving anticoagulation. Both scores performed poorly in our cohort, underscoring the need for a validated bleeding risk assessment model tailored to cancer patients. In contrast, using GDF-15 levels alone as a predictor resulted in significantly better discriminatory performance. Moreover, incorporating GDF-15 levels into the risk scores drastically improved their performance.

Our results were obtained from a heterogeneous group of consecutive patients, with 15% receiving anticoagulation therapy, making them more representative of the patients encountered in daily clinical practice rather than a highly selected trial population. Furthermore, our cohort reflects a population of cancer patients at high risk for VTE, treatment complications, and increased morbidity and mortality. Clear guideline recommendations exist for this group of ambulatory cancer patients undergoing systemic anticancer therapy, and some may be candidates for primary prevention strategies.^7^^,^^8^ However, the implementation of general primary thromboprophylaxis is hindered by the potential for increased bleeding risk.^30^

Assessing baseline bleeding risk is therefore crucial for identifying high-risk patients and including bleeding risk prediction into the decision-making process. Predicting baseline bleeding risk could aid in identifying patients who require closer monitoring and targeted interventions to mitigate specific risk factors, such as arterial hypertension, diabetes mellitus, antiplatelet drugs, or declining kidney function.

Notably, consistent with our previous study on GDF-15 in cancer,^20^ GDF-15 was not associated with VTE. Although a link with thrombotic outcomes has been reported in other contexts, this appears less relevant in the cancer setting, in which stronger risk factors likely play a more significant role. This distinction makes GDF-15 particularly promising, as many markers for bleeding also serve as markers for thromboembolism, complicating the differentiation between bleeding and thrombotic risk.

Longitudinal measurements of GDF-15 at 2 follow-up visits showed slight increases in GDF-15 levels over time, contrasting with observations in healthy individuals^31^ or patients with a steady disease state,^15^ in which levels remain more stable. This likely reflects the stress conditions cancer patients experience throughout their disease trajectory. Despite this, the association of GDF-15 levels and the future risk for major bleeding remained consistent and strong over time.

Although various time-varying factors (eg, thrombocytopenia, treatment regimen) that could increase bleeding risk were present in our cancer cohort, we focused on baseline predictors to maintain the integrity of predictive accuracy. Our findings show that GDF-15 levels remained strongly associated with bleeding risk, suggesting their robust predictive value even without accounting for additional time-dependent factors.

Not surprisingly, GDF-15 was also associated with all-cause mortality in our cohort. Previous studies in various disease settings have reported that elevated GDF-15 levels are independently associated with poor overall survival.^12^^,^^17^ Furthermore, GDF-15 has been incorporated into a risk score for predicting mortality in patients with AF.^12^^,^^17^^,^^32^ In the cancer population, the association between GDF-15 and overall survival has also been well documented,^20^ with reports spanning a wide variety of tumor subtypes, stages, and treatment settings.^12^

Interestingly, GDF-15 levels were substantially higher in patients with hepatobiliary cancer and correlated, to some extent, with markers of liver function. The exact mechanisms underlying these observations remain unclear. Ongoing research aims to uncover these mechanisms and leverage this understanding to explore potential novel treatment approaches.^12^

### Study limitations

First, the subgroup of anticoagulated patients in our GDF-15 cohort was relatively small, which may limit the generalizability of our findings for this specific patient group. However, extensive data on patients with AF and the small cancer patient cohort in the AVERT trial suggest that the association between GDF-15 and bleeding risk is at least as strong, if not stronger, in patients receiving anticoagulation compared with those not receiving anticoagulation.

Second, our study included patients from a single tertiary care center who were initiating systemic anticancer therapy, which may limit the applicability of our findings to the broader cancer population.

Third, the results of the HAS-BLED and CAT-BLEED analyses should be interpreted with caution, as neither score was designed for this specific setting. The internal validity of our study is supported by the moderate to large sample size, the large number of outcome events, and the robustness of our findings in longitudinal analysis of follow-up GDF-15 measurements. External validity is reinforced by the inclusion of an unselected, heterogenous cohort of consecutive cancer patients, reflecting the diversity of patients and tumor entities seen in daily clinical practice.

Fourth, our study could not provide insight into the mechanistic role of GDF-15 in bleeding risk. However, the general nonspecificity of GDF-15 may, in fact, enhance its value as a marker for bleeding events, which themselves are heterogeneous and arise from various causes in patients with cancer.

## Conclusions

In this study, we demonstrated that elevated GDF-15 levels in cancer patients are significantly and independently associated with an increased risk for major bleeding, even after adjusting for a wide range of clinical, cancer-specific, and laboratory parameters. Our analyses confirmed the strong predictive performance of GDF-15 in terms of both discrimination and calibration for bleeding risk. Importantly, the lack of association between GDF-15 and VTE underscores its specificity and potential utility as an optimal biomarker for bleeding risk prediction in cancer patients. These findings highlight the promise of GDF-15 as a valuable biomarker in the clinical assessment of bleeding risk, paving the way for more tailored and effective preventive strategies in this high-risk population.

Perspectives **COMPETENCY IN MEDICAL KNOWLEDGE:** GDF-15 levels are associated with major bleeding risk but not VTE in patients with cancer. GDF-15 also demonstrates predictive ability for major bleeding in this population.

**TRANSLATIONAL OUTLOOK:** Future studies should aim to unravel the mechanism underlying this association and validate the clinical utility of using GDF-15 for estimating bleeding risk in routine practice.

## Funding Support and Author Disclosures

This project was supported by an unrestricted grant from Roche Diagnostics International to the Medical University of Vienna and by the Society of Thrombosis and Hemostasis Research (Gesellschaft für Thrombose-und Hämostaseforschung) Early Career Research Grant 2021 to Dr Moik. Financial support from the Austrian Federal Ministry for Digital and Economic Affairs, the National Foundation for Research, Technology and Development, and the Christian Doppler Research Association is gratefully acknowledged. Dr Moik has received honoraria for lectures from Bristol Myers Squibb and Merck Sharpe & Dohme; and has participated on advisory boards for Bristol Myers Squibb. Dr Starzer has received honoraria for lectures from AstraZeneca; and has received travel and congress support from AstraZeneca, Stemline Menarini, PharmaMar, Merck Sharpe & Dohme, and Eli Lilly. Dr Preusser has received honoraria for lectures, consultation, or advisory board participation from Bayer, Bristol Myers Squibb, Novartis, Gerson Lehrman Group, CMC Contrast, GlaxoSmithKline, Mundipharma, Roche, BMJ Journals, MedMedia, AstraZeneca, AbbVie, Eli Lilly, Med Ahead, Daiichi-Sankyo, Sanofi, Merck Sharpe & Dohme, Tocagen, Adastra, Gan & Lee Pharmaceuticals, Janssen, Servier, Miltenyi Biotec, and Boehringer-Ingelheim. Dr Berghoff has received research support from Daiichi-Sankyo and Roche; has received honoraria for lectures, consultation, or advisory board participation from Roche, Bristol Meyers Squibb, Merck, Daiichi-Sankyo, AstraZeneca, CeCaVa, and Seagen; and has received travel support from Roche, Amgen, and AbbVie. Dr Pabinger has received personal fees for lectures and/or participation on advisory boards from Bristol Myers Squibb/Pfizer, Daiichi-Sankyo, Rovi, and Sanofi. Dr Ay has received personal fees for lectures and/or participation on advisory boards from Bayer, Bristol Myers Squibb/Pfizer, Daiichi-Sankyo, and Sanofi. All other authors have reported that they have no relationships relevant to the contents of this paper to disclose.
