# Supplementary material for: Growth Differentiation Factor-15 Predicts Major Bleeding in Cancer Patients: Results From the Vienna CAT-BLED Study
Source: JACC CardioOncol. 2025 Jan 14;7(2):141–52. doi: 10.1016/j.jaccao.2024.11.007 (PMC11866448; doi:10.1016/j.jaccao.2024.11.007)
Supplement: Supplemental Material [file mmc1.docx]

**Supplement:
Growth differentiation factor-15 predicts major bleeding in cancer patients: results from the Vienna CAT-BLED study**

Cornelia Englisch^1*^, Stephan Nopp^1*^, Ingrid Pabinger^1^, Florian Moik^1,2^, Daniel Steiner^1^, Angelika M. Starzer^3,4^, Monika Fritzer-Szekeres^5^, Matthias Preusser^3,4^, Anna S. Berghoff^3,4^, Cihan Ay^1^

*contributed equally as first authors

^1^Division of Hematology and Hemostaseology, Department of Medicine I, Medical University of Vienna; Vienna, Austria

^2^Division of Oncology, Department of Internal Medicine, Medical University of Graz; Graz, Austria

^3^Division of Oncology, Department of Medicine I, Medical University of Vienna; Vienna, Austria

^4^Christian Doppler Laboratory for Personalized Immunotherapy, Department of Medicine I, Medical University of Vienna, Vienna, Austria

^5^Department of Laboratory Medicine, Medical University of Vienna; Vienna, Austria

**Table of Contents**

**Statistical analysis plan3**

**Supplementary figure 18**

**Supplementary figure 29**

**Supplementary figure 310**

**Supplementary figure 411**

**Supplementary figure 512**

**Supplementary figure 613**

**Supplementary figure 714**

**Supplementary figure 815**

**Supplementary figure 916**

**Supplementary figure 1017**

GDF-15 in patients with cancer

-

Statistical analysis plan

Template provided by Prof. Georg Heinze

# 1 Administrative information

| Title of study | Evaluating Growth Differentiation Factor-15 as a Biomarker for Major Bleeding Risk in Cancer Patients |
| --- | --- |
| SAP version | 1.1 – 16.07.2023 |
| Roles and responsibilities | Stephan Nopp^1^: writing of the statistical analysis plan  Cornelia Englisch^1^: writing of the statistical analysis plan  ^1^ Department of Medicine I, Medical University of Vienna, Vienna, Austria |
| Signatures | 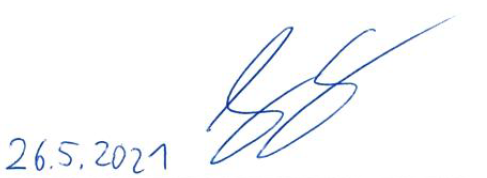 |

# 2 Data management

| Data management plan available as separate document | discussed in separate file |
| --- | --- |

# 3 List of variables

*List all variables which are contained in the study data base, including derived variables (e.g. body-mass-index).*

| **primary outcome variable(s)** | | **short variable name** | **time point** | **scale of measurement or levels** |
| --- | --- | --- | --- | --- |
| Major Bleeding | | MB | Within 2-year follow-up period | Nominal, (Yes/No) |
| Time to major bleeding | | Time2mb | Within 2-year follow-up period | Metric (days) |
| **secondary outcome variable(s)** | **short variable name** | | **time point** | **scale of measurement or levels** |
| Death | death | | Within 2-year follow-up period | Nominal, (Yes/No) |
| Survival time | Time2death | | Within 2-year follow-up period | Metric (days) |

| **study specific variable(s)** | **short variable name** | **time point** | **scale of measurement or levels** |
| --- | --- | --- | --- |
| GDF-15 | gdf | T0, T1, T2 | Metric (ng/L) |

| **further subject characteristics** | **short variable name** | **time point** | **scale of measurement or levels** |
| --- | --- | --- | --- |
| Age | age | T0 | Metric (years) |
| Sex | sex | T0 | nominal, 2 levels  (female, male) |
| BMI | bmi | T0 | Metric (kg/m²) |
| Stage IV disease | stage | T0 | Nominal, 2 levels (yes, no) |
| Type of cancer | type | T0 | Nominal, 15 levels  (brain, breast, esophageal, stomach, colorectal, prostate, gynecological, sarcoma, lymphoma, urinary, head&neck, pancreas, lung, hepatobiliary, others) |
| Newly diagnosed cancer | newdiagnosis | T0 | Nominal, (Yes/No) |
| Type of cancer treatment | treatment | T0 | Nominal, 9 levels (more than 1 is possible)  (immunotherapy, chemotherapy, chemoimmunotherapy, targeted therapy, target&chemotherapy, TKI therapy, non-systemic therapy, targeted&immunotherapy, other) |
| Anticoagulation | AC | T0 | Nominal, 2 levels (yes, no) |
| Antiplatelet | AP | T0 | Nominal, 2 levels (yes, no) |

# 4 Initial data analysis

| **Descriptive univariate analyses** | **Variable** | **Analyses** | | |
| --- | --- | --- | --- | --- |
| GDF-15 | gdf | Summary statistics and visualization via histogram | | |
| **Bivariate analyses of non-outcome variables** | **Variable 1** | **Variable 2** | **Analysis** | |
| Correlation of GDF-15 and age | gdf | age | Correlation analysis of GDF-15 and age | |
| Distribution of GDF-15 stratified by sex | gdf | sex | Summary statistics stratified by sex | |
| Correlation of GDF-15 and BMI | gdf | BMI | Correlation analysis of GDF-15 and BMI | |
| Distribution of GDF-15 stratified by tumor stage | gdf | stage | Summary statistics stratified by tumor stage | |
| Distribution of GDF-15 across different tumor types/sites | gdf | type | Summary statistics stratified by tumor type/site | |
| Distribution of GDF-15 across AC/no AC | gdf | AC | Summary statistics stratified by tumor type/site | |
| **Trivariate analyses of non-outcome variables** | **Variable 1** | **Variable 2** | **Variable 3** | **Analysis** |
| Distribution of GDF-15 stratified by age and sex | gdf | age | sex | Summary statistics stratified by age and sex |
| Distribution of GDF-15 stratified by age and bmi | gdf | age | bmi | Summary statistics stratified by age and bmi |
| Distribution of GDF-15 stratified by sex and bmi | gdf | sex | bmi | Summary statistics stratified by sex and bmi |
| Distribution of GDF-15 stratified by sex and tumor type | gdf | sex | type | Summary statistics stratified by sex and tumor type |
| Distribution of GDF-15 stratified by sex and tumor stage | gdf | sex | stage | Summary statistics stratified by sex and tumor stage |
| Distribution of GDF-15 stratified by stage and tumor type | gdf | type | stage | Summary statistics stratified by tumor type and stage |
| Distribution of GDF-15 stratified by AC and tumor type | gdf | AC | type | Summary statistics |
| Distribution of GDF-15 stratified by AC and tumor type | gdf | AC | stage | Summary statistics |
|  |  |  |  |  |
| **Missing values** | Number and proportion of missing values per variable | | | |
| **Patterns of missing values** | Grouping of subjects where the same variables are systematically missing  Grouping of variables which are missing simultaneously | | | |

**Possible consequences from initial data analysis**

| **Condition** | **Consequence** |
| --- | --- |
| Missing values in one variable of 40% and more | Exclusion of variable from analysis |
| Missing values in GDF-15 | Exclusion of patient |
| skew distribution of continuous variables | log-transformation to avoid disproportional impact of outliers |

# 5 Descriptive analysis of study group

The descriptive statistics of the study participants' baseline characteristics will be presented in a detailed table. This includes variables such as Age, Sex, BMI, Tumor Stage, Tumor Type, Cancer Treatment planned during the observation period, among others.

For categorical variables, both absolute and relative frequencies will be described. Continuous variables, on the other hand, will be represented by medians along with their 25th and 75th percentiles.

Cumulative incidence of major bleeding will be estimated with cause-specific cumulative incidence estimators, treating death not related to bleeding as a competing event.

# 6 Main analysis methods

A complete case analysis will be performed in this study.

**Primary analysis:**

The association between GDF-15 levels and risk of major bleeding will be evaluated using a Fine and Gray proportional subdistribution hazards regression model. Mortality will be accounted for as a competing risk. We will adjust for several covariates in a stepwise fashion and present adjusted subdistribution hazard ratios. The fully adjusted model will include GDF-15, age, sex, and BMI, tumor stage, tumor type, laboratory parameters (hemoglobin, creatinine, albumin) and antithrombotic therapies. Covariates are chosen based on known risk factors for bleeding and from findings from our previous study on GDF-15 (Nopp et al., JTH 2023).

Moreover, it will be assessed if GDF-15 can predict major bleeding occurrence in the next 6-months after measurement. The prognostic value of GDF-15 for major bleeding risk prediction will be evaluated compared to established bleeding risk scores, i.e., HAS-BLED, CAT-BLEED. Discrimination will be measured using the Harrel’s C-index. The C-index will be calculated using the rms package in R by including all observational periods of each patient. 95% confidence intervals will be reported from bootstrapped values with 500 replicates, where patients are resampled with replacement.

Similarly, calibration will be evaluated by computing the observed/expected ratio and the calibration slope by fitting a Fine-Gray model with the linear predictor from the original model as single variable. 95% confidence intervals will be similarly computed.

Addition of GDF-15 to those scores will be assessed using measures of model comparison including net reclassification index (NRI), integrated discrimination improvement (IDI), likelihood-ratio testing, adequacy index, and fraction of new information (similar to the previous publication of a different cohort; Nopp et al. JTH, 2023).

All statistical will be done in R Studio (v4.2.0; R Studio, Vienna, Austria).


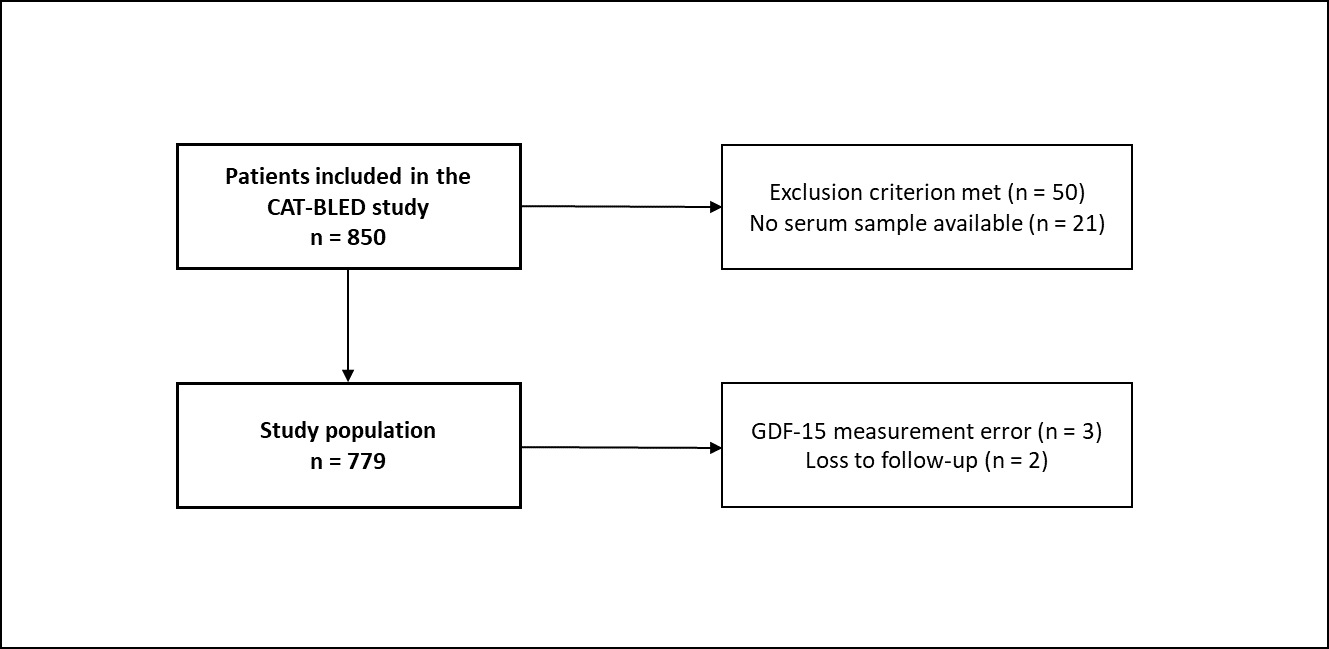


**Supplementary figure 1. Study flow chart.** CAT-BLED - Vienna Cancer, Thrombosis and Bleeding study


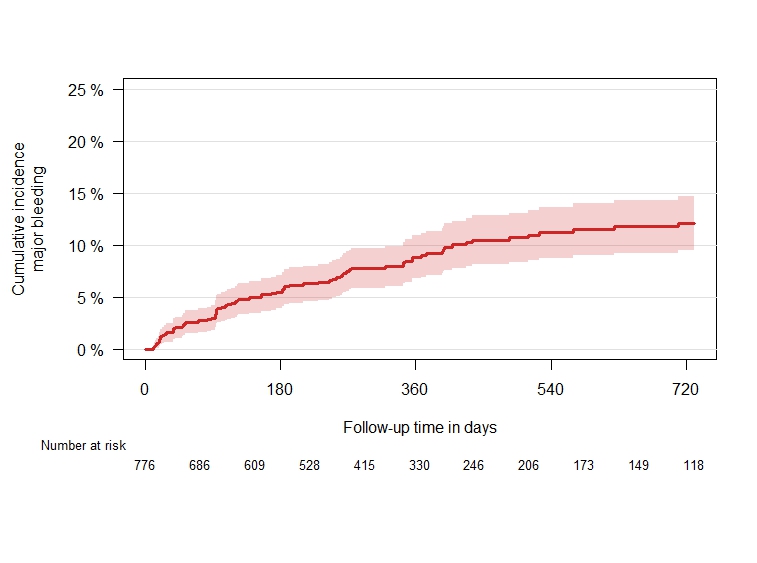


**Supplementary figure 2. Cumulative major bleeding incidence in the CAT-BLED study during two years of follow-up.**


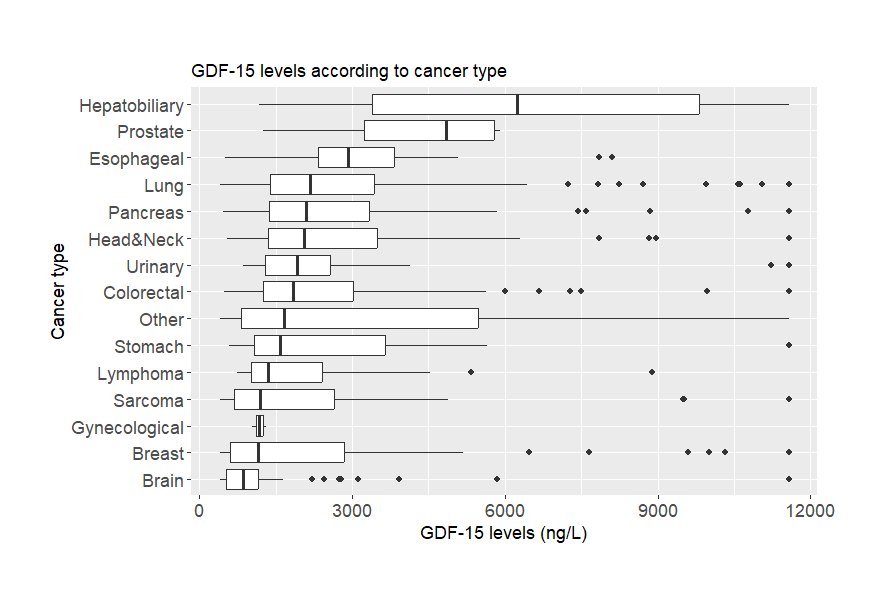


**Supplementary figure 3. GDF-15 levels at study inclusion according to cancer type.** For the x-axis the 95^th^ percentile of GDF-15 was used as a cut-off. Bold line represents median; upper and lower hinge represent third and first quartile, respectively; points indicate outliers.


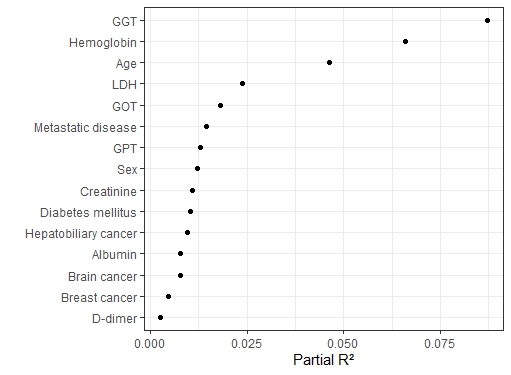


**Supplementary figure 4. Association between growth differentiation factor-15 (GDF-15) and variables measured at study inclusion**. Patient demographics (age, sex, BMI), tumor specific variables (metastatic disease, tumor type), comorbidities (arterial hypertension, atrial fibrillation, diabetes mellitus), and laboratory values (albumin, hemoglobin, D-dimer, creatinine, platelets, leukocytes, CRP, LDH, GGT, GOT, GPT) have been tested and the top 15 variables are displayed. The partial R² value represents the amount each variable contributes to explaining the total variance of GDF-15 at baseline. No variable explains more than 10% of variation and the total R² value is below 0.50.


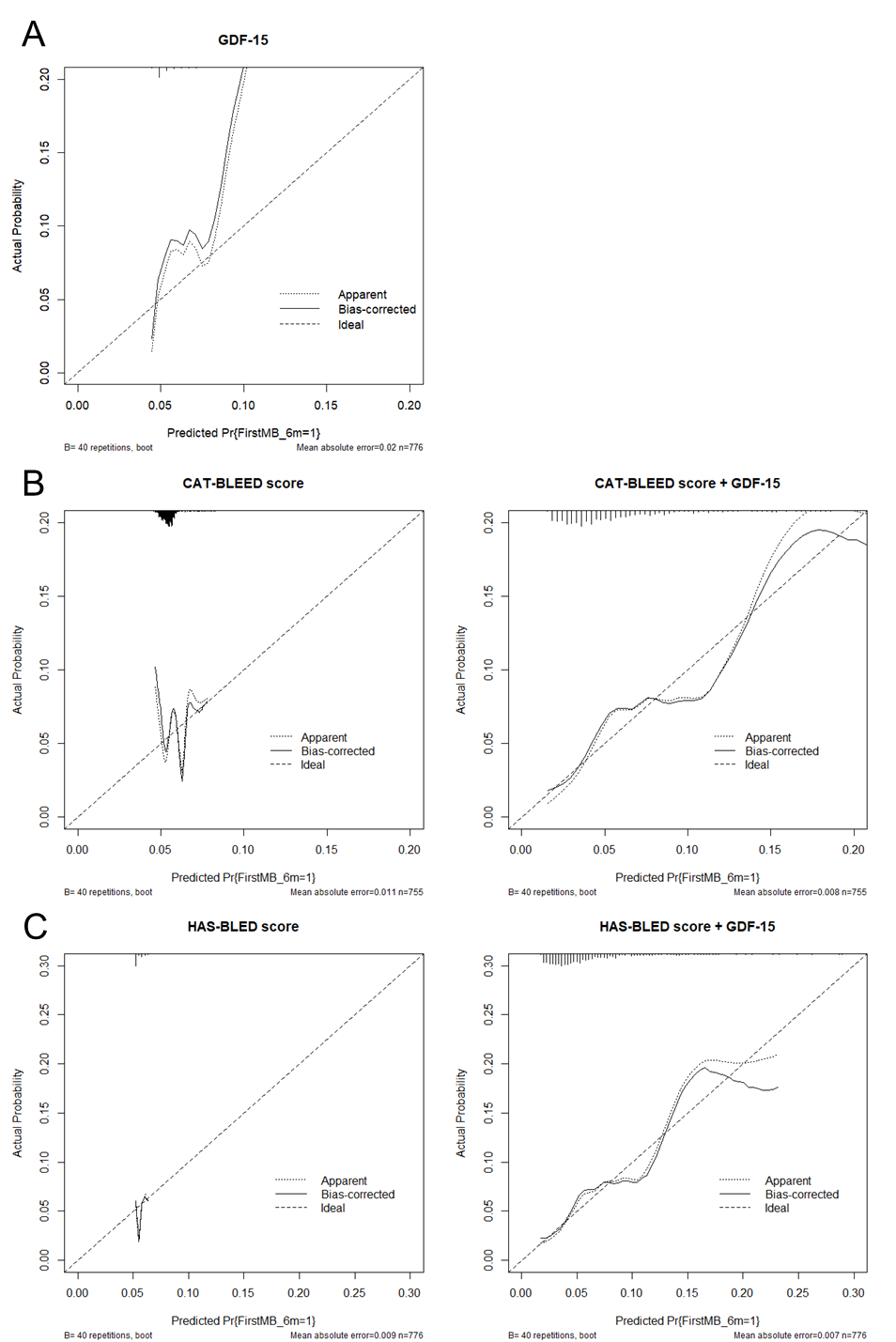


Supplementary figure 5. Calibration plots for predicting major bleeding using only GDF-15 (A), the CAT-BLEED (B), and HAS-BLED score (C) with and without GDF-15.

**
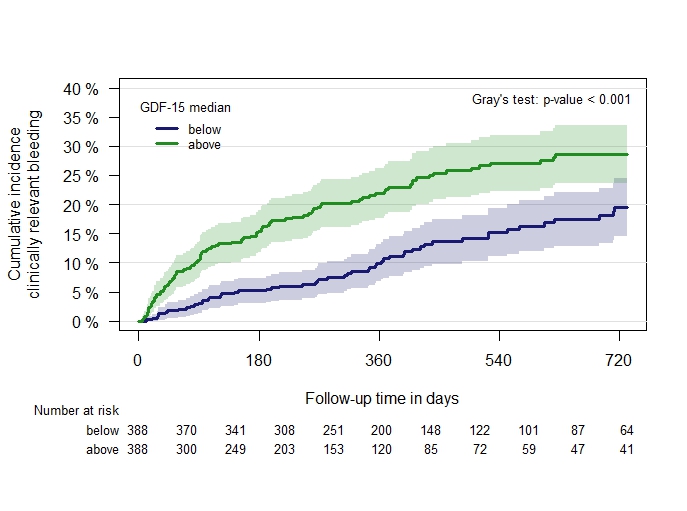
**

Supplementary figure 6. Cumulative clinically relevant bleeding incidence of patients with growth differentiation factor-15 (GDF-15) levels above versus below the cohort median. Patients were stratified according to their GDF-15 level and the group with levels above 1864 ng/L (>median) was compared to the group with levels below 1864 ng/L (≤median) using Gray's test for competing risks.

**
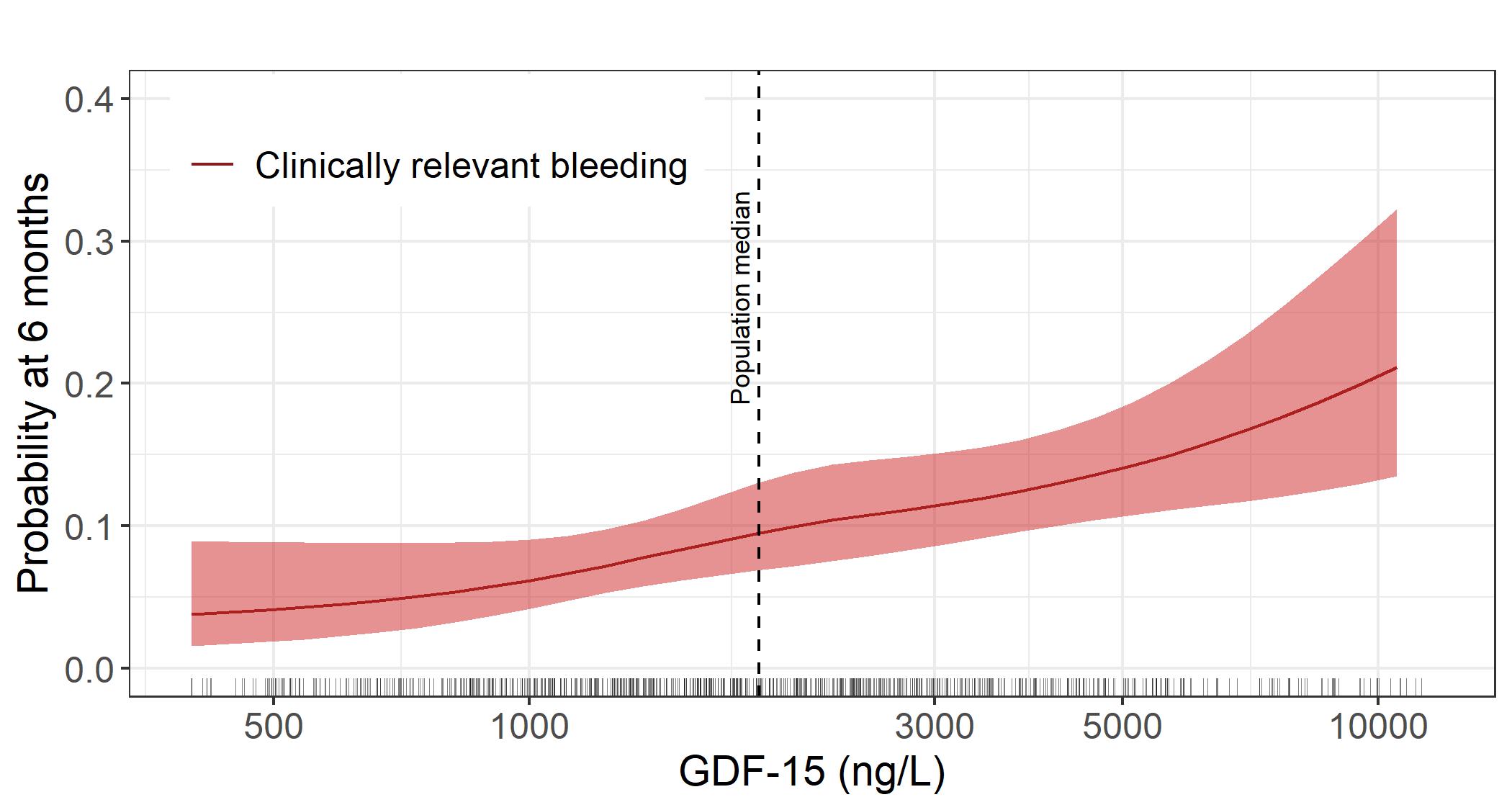
**

Supplementary figure 7. Six-month predicted probabilities of clinically relevant bleeding in relation to continuous growth differentiation factor-15 (GDF-15) levels. The x-axis was log10-transformed. Predicted probabilities are shown up to the 95th percentile of GDF-15 levels, which in total ranges from 400 to 59,707 ng/L. The dashed line represents the cohort median of GDF-15 levels.

**Supplementary figure 8. Association of growth differentiation factor-15 (GDF-15) with clinically relevant bleeding in uni- and multivariable analyses.** Subdistribution hazard ratios (SHR) are displayed for log2-transformed levels of GDF-15. The hazard ratio can be interpreted as the risk increase by doubling of GDF-15 levels. All-cause death was considered a competing risk. Patients with head and neck cancer were deemed at high risk for bleeding and, thus, adjustment for head and neck versus other was performed.

**
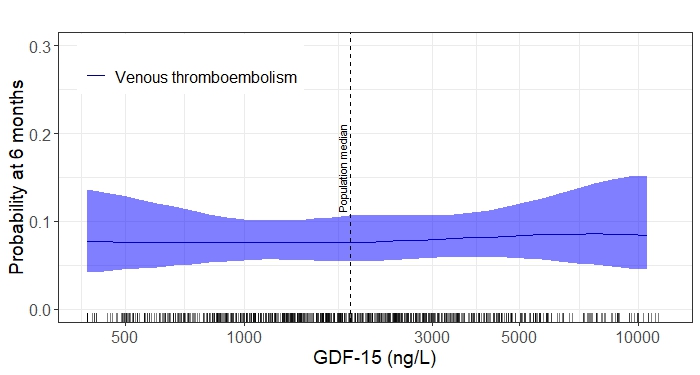
**

Supplementary figure 9. Six-month predicted probabilities of venous thromboembolism in relation to continuous growth differentiation factor-15 (GDF-15) levels. The x-axis was log10-transformed. Predicted probabilities are shown up to the 95th percentile of GDF-15 levels, which in total ranges from 400 to 59,707 ng/L. The dashed line represents the cohort median of GDF-15 levels.

**Supplementary figure 10. Association of growth differentiation factor-15 (GDF-15) with venous thromboembolism in uni- and multivariable analyses.** Subdistribution hazard ratios (SHR) are displayed for log2-transformed levels of GDF-15. The hazard ratio can be interpreted as the risk increase by doubling of GDF-15 levels. All-cause death was considered a competing risk. Patients with pancreatic, gastroesophageal, and brain cancers were classified as high risk, while those with breast and prostate cancers were classified as low risk. Adjustments for tumor type were made accordingly in the analysis.
